# Trends in migraine incidence among women of childbearing age from 1990 to 2019 and the prediction for 2030: an analysis of national data in China

**DOI:** 10.1186/s10194-023-01692-0

**Published:** 2023-11-27

**Authors:** Zhuanzhuan Fan, Jian Kang, Wenting Li, Zhiyong Wang, Huifen Qiao, Fei Xu

**Affiliations:** 1 Nanjing Municipal Center for Disease Control and Prevention Affiliated to Nanjing Medical University, Nanjing, China; 2 The First Affiliated Hospital With Nanjing Medical University, Nanjing, China; 3 Nanjing Brain Hospital Affiliated to Nanjing Medical University, Nanjing, China; 4 Nanjing Medical University School of Public Health, Nanjing, China

**Keywords:** Migraine, Incidence, Women, Childbearing age, Chinese

## Abstract

**Background:**

Migraine is a primary headache, which has been producing heavy disease burden globally. There is no data on the incidence of migraine among women of childbearing age worldwide, including China. This study aimed to investigate the time trend in incidence rate of migraine among women of childbearing age in China from 1999 to 2019, and to make a prediction for 2030.

**Methods:**

Data on migraine incidence and population among women of childbearing age in China were derived from the Global Burden of Diseases Study 2019. Crude and age-standardized incidence rates of migraine (CIR, ASIR) were presented. The trend in migraine during 1990–2019 was examined using annual percent change and average annual percent change based on Joinpoint regression models. Age-period-cohort model was introduced to estimate the independent effect of age, period and cohort on migraine incidence rate among participants over the three decades. Bayesian age-period-cohort analysis was conducted to predict migraine incidence rate for 2030 among women of childbearing age in China.

**Results:**

For women of childbearing age in China, the case number, CIR and ASIR of migraine kept rising, with a cumulative percentage increase of 10.87%, 2.01% and 5.65%, respectively, from 1990 to 2019. An annual percent increase of 0.18% in the ASIR was observed over the three decades. As for the age, period and cohort effects, the adjusted cohort-specific relative risks constantly increased from 0.91 (95% CI: 0.90, 0.93) in the 1940–1949 cohort to 1.04 (95% CI: 1.03, 1.05) in the 1995–2004 cohort, while the period-specific relative risks initially declined from 1.00 (95% CI: 0.99, 1.00) in 1990–1994 cohort to 0.99 (95% CI: 0.98, 0.99) in 1995–1999 cohort, and then increased to 1.04 (95% CI: 1.03, 1.04) in 2015–2019 cohort. Moreover, the age-specific relative risks of migraine followed a bimodal pattern with peaks at the age-group of 25–29 years (CIR = 1718.27/100000) and 35–39 years (CIR = 1635.18/100000). Projection modeling showed that the CIR and ASIR of migraine will continue to significantly increase from 2020 to 2030.

**Conclusion:**

Migraine incidence remained an increasing trend from 1990 to 2019 and is projected to continually increase till 2030 among women of childbearing age in China. This study has important public health implication for population-level migraine prevention in China. Precision intervention strategies and approaches shall be considered in campaigns initiated for migraine prevention among Chinese women of childbearing age.

## Background

Migraine is a primary headache, typically characterized by recurrent moderate or severe unilateral pulsatile headaches [1]. It has been well documented that migraine had a detrimental impact on individual’s physical and emotional well-beings, including the elevated risk of stroke, cardiovascular diseases, anxiety, and other mental problems [2–5]. Moreover, migraine may also cause economic and social issues such as financial costs, reduced educational attainment, impaired academic performance, and diminished economic productivity [6, 7].

Migraine has been causing heavy disease burden worldwide. About 15% of world population may experience migraine per year and migraine also ranked the second leading cause of disability globally [8, 9]. China is one of the most populous countries in the world, with an average migraine prevalence of 7.9%—14.3% in adult population [10, 11], implying that there were approximately 0.11 to 0.20 billion adults affected by migraine. Due to having the largest population of migraine sufferers, China now bears the most significant health, economic and social burden caused by migraine in the world [9].

Women, especially those of childbearing age (15–49 years), than men not only were more vulnerable to suffering from migraine but also would need longer time to recover from an attack [12, 13]. Furthermore, due to extra physiological and psychological demands, women of childbearing age tend to experience migraine [8, 12] and were more easily to be affected by migraine on their career development even compared to those women aged 49 + years [14]. Additionally, migraine is the leading cause of Disability Adjusted of Life Years (DALYs) in women of childbearing age [8], and moreover menstrual migraine and chronic migraine may affect their pregnancy plans [15]. Therefore, it deserves to assess the burden caused by migraine among women of childbearing age for initiating precision intervention campaigns in China.

Previous studies in China documented the prevalence of migraine in overall population or medical college students based on cross-sectional surveys [16–18]. However, there is no study reporting migraine prevalence, particularly incidence, among Chinese women of childbearing age so far. Thus, this study aimed to address knowledge gaps by investigating the time trend of nationwide migraine incidence rate for the period between 1990 and 2019, examining effects of age, period and cohort on migraine incidence during the study period, and further predicting migraine incidence for 2030.

## Materails and methods

### Data sources

Data on migraine and population analyzed in the present study were from The Global Burden of Diseases, Injuries, and Risk Factors study 2019 (GBD 2019 study) [1, 19, 20], which were freely available from the Global Health Data Exchange (https://ghdx.healthdata.org/gbd-resultstool) (Data Source: Institute for Health Metrics and Evaluation. Used with permission. All rights reserved.). Details on methodology, statistical approaches and metrics of the GBD 2019 study have been reported in details elsewhere [1, 19, 20]. In GBD 2019 study, data on migraine in China were estimated based on published population-based incidence studies [1]. Specific data were derived from the GBD database using strategies of assigning "China" as the location, "Migraine" as the cause, and "Incidence" as the measure [21]. Due to that all information had been de-identified and publicly available, ethical approval was exempted for our study.

### Participants and variables

Migraine was identified according to the International Classification of Headache Disorders, 3^rd^ Edition (ICHD-3) in the GBD 2019 study. An individual was defined as a migraine patient, if she met all the five criteria of ICHD-3 classification: 1) at least five attacks that meeting criteria 2–5; 2) each headache attack lasting for 4–72 h (untreated or treated unsuccessful); 3) headache being with at least two of the following four features (a. unilateral localization, b. pulsating quality, c. moderate or severe pain intensity, and d. aggravation by or causing avoidance of routine physical activity); 4) during each headache attack, at least one of the following symptoms experienced (a. nausea and/or vomiting, b. photophobia and phonophobia); and 5) not better accounted for by another ICHD-3 diagnosis[1]. In this study, migraine was coded as G43-G43.919 in the International Classification of Diseases, the tenth Revision (ICD-10). Additionally, to ensure comparability to existing studies, aura was not considered in this study, as it was usually not assessed in previous epidemiological investigations regarding migraine [1].

For this study, eligible participants were those Chinese women of childbearing age and outcome event was migraine case recorded in the GBD dataset during the period between 1990 and 2019. Meanwhile, women of childbearing age were classified into age-specific categories with a five-year interval in the analysis: 15–19, 20–24, 25–29, 30–34, 35–39, 40–44 and 45–49 years.

### Outcome measures

Outcome measures were case number, crude incidence rate (CIR) and age-standardized incidence rate (ASIR) of migraine. And, then, annual percent change (APC) and average annual percent change (AAPC) were used to examine the temporal trends in the CIR and ASIR of migraine.

### Statistical analysis

Firstly, descriptive analysis was performed to present epidemiological distribution of migraine incidence among participants by selected personal characteristics, using the statistical program R (version 4.1.3) with the packages "tidyverse" and "ggplot2". Then, APC and AAPC were calculated using Joinpoint Regression Program software (version 4.9.0.1, Statistical Research and Applications Branch, National Cancer Institute, USA) to investigate the temporal trend of migraine incidence over the three decades. The hypothesis test was implemented to determine whether the AAPC/APC significantly deviated from zero. APC/AAPC > 0 means an increasing trend, while APC/AAPC < 0 means a decreasing trend during the segment [22].

Next, age-period-cohort model was introduced to compute relative risks (RRs) for assessing the effects of age, period and cohort on incidence of migraine. The model was based on a log-linear model for estimating the rate with additive effects of age, calendar time period and birth cohort, as shown in the following formula [23]:

$$\mathrm{Log\lambda ij }=\upmu +\mathrm{ \alpha i }+\mathrm{ \beta j }+\mathrm{ \gamma k i }= 1, 2, . . . ,\mathrm{ I}$$

$$\mathrm{j }= 1, 2, . . . ,\mathrm{ J}$$

$$\mathrm{k }=\mathrm{ j }-\mathrm{ i }+\mathrm{ I}$$

In this model, µ refers to the intercept term, αi refers to the age effects, βj refers to the period effects, and γki refers to the cohort.

Finally, Bayesian age-period-cohort model, utilizing the R packages "BAPC", was employed to predict the case number, CIR, and ASIR of migraine from 2020 to 2030. In this model, the estimated population of China from 2020 to 2030 was also obtained from Global Health Data Exchange website (https://ghdx.healthdata.org/record/ihme-data/global-population-forecasts-2017-2100, accessed on: April 20, 2023).

## Results

### Migraine case and incidence between 1990 and 2019

Table 1 presented the case number and incidence rate of migraine among women of childbearing age in China during 1990 and 2019. Overall, there were 493.31 × 10^**4**^ and 546.94 × 10^**4**^ migraine cases in the year of 1990 and 2019, separately, among women of childbearing age in China. Meanwhile, CIR and ASIR were, separately, 1527.16/100000 and 1505.25/100000 in 1990, and 1557.81/100000 and 1590.29/100000 in 2019. Over the three decades, a cumulative increase in CIR and ASIR were 2.01% and 5.65%, respectively, among the study women. Interestingly, the highest crude incidence rate of migraine was observed in women aged 25–29 years across the three decades, although the change in CIR of migraine was not the highest for women within this age-group.

**Table 1:** Cases and incidence rates of migraine among women of childbearing age in China 1990–2019

Year	Measures	Women of childbearing age							
		Overall	15–19 years	20–24 years	25–29 years	30–34 years	35–39 years	40–44 years	45–49 years
	Cases^a^	493.31	96.33	99.95	90.58	66.58	68.9	44.69	26.28
1990	CIR^b^	1527.16	1560.67	1544.63	1688.59	1571.88	1559.2	1397.34	1076.09
	ASIR^c^	1505.25	N/A	N/A	N/A	N/A	N/A	N/A	N/A
	Cases^a^	533.77	76.94	75.02	101.65	100.36	77.52	59.11	61.22
1999	CIR^b^	1491.62	1522.3	1506.57	1677.8	1559.31	1547.97	1400.69	1519.55
	ASIR^c^	1487.64	N/A	N/A	N/A	N/A	N/A	N/A	N/A
	Cases^a^	604.52	78.43	102.79	90.09	79.59	97.97	94.6	61.04
2010	CIR^b^	1539.59	1580.14	1567.52	1732.72	1614.68	1643.55	1498.47	1142.91
	ASIR^c^	1556.77	N/A	N/A	N/A	N/A	N/A	N/A	N/A
	Cases^a^	546.94	58.83	62.97	96.08	104.66	81.37	74.83	68.2
2019	CIR^b^	1557.81	1678.92	1610.47	1767.49	1640.39	1643.88	1503.74	1145.59
	ASIR^c^	1590.29	N/A	N/A	N/A	N/A	N/A	N/A	N/A
	Change in cases (%)	10.87	-38.93	-37	6.07	57.19	18.1	67.44	159.51
1990–2019	Change in CIR (%)	2.01	7.58	4.26	4.67	4.36	5.43	7.61	6.46
	Change in ASIR (%)	5.65	N/A	N/A	N/A	N/A	N/A	N/A	N/A

^a^Cases: the number of migraine cases (× 10^4^)

^a^*CIR* Crude incidence rate (/100,000)

^c^*ASIR* Age-standardized incidence rate (/100,000)

### The temporal trend in migraine incidence over the three decades

Table 2 showed the temporal trend in migraine incidence among women of childbearing age in China 1990–2019. Among the overall participants, five time periods were identified and the APC of ASIR was significant for each time period, while, on average, the ASIR of migraine increased by 0.18% (95%CI: 0.17%, 0.20%) per year during the three decades. Additionally, four patterns were examined regarding the temporal trend of migraine ASIR over the 30 years. The first, a declining trend of ASIR was observed in period 1 (1990–1996) with an APC of -0.02 (95% CI: -0.05, -0.00), and period 2 (1996–2000) with an APC of -0.35 (95% CI: -0.42, -0.29). The second, a sharply escalating trend in ASIR was examined in period 3 (2000–2005) with an APC of 1.03% (95% CI: 0.99, 1.07). The third, a slight decrease in ASIR was identified in period 4 (2005–2017) with an APC of -0.03 (95%CI: -0.04, -0.02). Then, ASIR rose again in period 5 (2017–2019) with an APC of 1.06 (95%CI: 0.93, 1.20). With regard to AAPC of CIR across different age-groups of subjects, similar patterns were observed over the three decades (Table 3).

**Table 2:** Temporal trends in migraine incidence rate among women of childbearing age in China 1990–2019^a^

Measures	Age groups of participants	Time periods identified		Temporal trend							
				APC^b^			AAPC^d^				
				%	95%CI^c^	P value	%	95% CI		*P* value	
ASIR	Overall	Period1	1990–1996	-0.02	(-0.05, -0.00)	0.05					
		Period2	1996–2000	-0.35	(-0.42, -0.29)	< 0.001					
		Period3	2000–2005	1.03	(0.99, 1.07)	< 0.001	0.18		(0.17, 0.20)		< 0.001
		Period4	2005–2017	-0.03	(-0.04, -0.02)	< 0.001					
		Period5	2017–2019	1.06	(0.93, 1.20)	< 0.001					
CIR		Period1	1990–1995	-0.13	(-0.17, -0.08)	< 0.001					
		Period2	1995–2000	-0.47	(-0.52, -0.41)	< 0.001					
	Overall	Period3	2000–2005	1.04	(0.98, 1.09)	< 0.001					
		Period4	2005–2012	-0.27	(-0.30, -0.24)	< 0.001	0.06		(0.04, 0.08)		< 0.001
		Period5	2012–2017	-0.01	(-0.07, 0.05)	0.71					
		Period6	2017–2019	0.79	(0.62, 0.97)	< 0.001					
		Period1	1990–1996	-0.04	(-0.11, 0.03)	0.25					
		Period2	1996–2000	-0.77	(-0.97, -0.56)	< 0.001					
	15–19 years	Period3	2000–2004	1.25	(1.05, 1.46)	< 0.001	0.24		(0.19, 0.28)		< 0.001
		Period4	2004–2017	-0.05	(-0.08, -0.03)	< 0.001					
		Period5	2017–2019	2.98	(2.56, 3.40)	< 0.001					
		Period1	1990–1996	-0.05	(-0.08, -0.02)	0.01					
		Period2	1996–2000	-0.73	(-0.82, -0.65)	< 0.001					
	20–24 years	Period3	2000–2005	0.88	(0.83, 0.94)	< 0.001	0.13		(0.12, 0.15)		< 0.001
		Period4	2005–2017	0.02	(0.004, 0.03)	0.01					
		Period5	2017–2019	1.25	(1.08, 1.44)	< 0.001					
		Period1	1990–1996	-0.02	(-0.03, -0.004)	0.02					
		Period2	1996–2000	-0.19	(-0.23, -0.15)	< 0.001					
	25–29 years	Period3	2000–2005	0.92	(0.89, 0.94)	< 0.001					
		Period4	2005–2009	-0.26	(-0.30, -0.22)	< 0.001	0.15		(0.14, 0.16)		< 0.001
		Period5	2009–2017	-0.02	(-0.03, -0.01)	0.001					
		Period6	2017–2019	0.97	(0.89, 1.05)	< 0.001					
		Period1	1900–1996	-0.02	(-0.04, -0.01)	0.01					
		Period2	1996–2000	-0.25	(-0.29, -0.20)	< 0.001					
	30–34 years	Period3	2000–2005	1.08	(1.05, 1.11)	< 0.001					
		Period4	2005–2009	-0.37	(-0.42, -0.33)	< 0.001	0.14		(0.13, 0.15)		< 0.001
		Period5	2009–2017	-0.02	(-0.03, -0.01)	0.01					
		Period6	2017–2019	0.77	(0.67, 0.87)	< 0.001					
		Period1	1990–1996	0.001	(-0.01, 0.02)	0.94					
		Period2	1996–2000	-0.27	(-0.31, -0.23)	< 0.001					
	35–39 years	Period3	2000–2005	1.04	(1.01, 1.06)	< 0.001	0.18		(0.17, 0.19)		< 0.001
		Period4	2005–2009	0.29	(0.25, 0.33)	< 0.001					
		Period5	2009–2019	0.01	(-0.001, 0.01)	0.1					
		Period1	1990–1998	0.01	(-0.02, 0.03)	0.58					
		Period2	1998–2001	0.26	(0.07, 0.45)	0.01					
	40–44 years	Period3	2001–2004	1.43	(1.24, 1.62)	< 0.001	0.25		(0.22, 0.28)		< 0.001
		Period4	2004–2008	0.44	(0.35, 0.54)	< 0.001					
		Period5	2008–2019	0.03	(0.02, 0.04)	< 0.001					
		Period1	1990–2001	-0.01	(-0.04, 0.02)	0.34					
	45–49 years	Period2	2001–2004	2.2	(1.74, 2.66)	< 0.001	0.21		(0.16, 0.25)		< 0.001
		Period3	2004–2019	-0.03	(-0.05, -0.01)	0.01					

^a^Temporal trends of migraine incidence were analyzed using joinpoint regression models

^b^*APC* Annual percent change

^c^*CI* Confidence interval

^#^*AAPC* Average annual percent change

**Table 3:** Estimated age, period and cohort effects of migraine among women of childbearing age in China 1990–2019

Factors	Net drift^a^, % per year	Local drift^b^	RR(95%CI)
Overall	0.21(0.19,0.23)		
Age			
15 to 19		0.15(0.10,0.19)	0.90(0.90,0.91)
20 to 24		0.12(0.09,0.15)	0.90(0.89,0.91)
25 to 29		0.14(0.12,0.17)	1.00
30 to 34		0.18(0.15,0.20)	0.94(0.94,0.94)
35 to 39		0.24(0.21,0.27)	0.95(0.95,0.96)
40 to 44		0.32(0.28,0.35)	0.88(0.87,0.88)
45 to 49		0.34(0.29,0.39)	0.68(0.68,0.69)
Period			
1990 to 1994			1.00(0.99,1.00)
1995 to 1999			0.99(0.98,0.99)
2000 to 2004			1.00
2005 to 2009			1.03(1.03,1.04)
2010 to 2014			1.03(1.03,1.04)
2015 to 2019			1.04(1.03,1.04)
Cohort			
1940 to 1949			0.91(0.90,0.93)
1945 to 1954			0.92(0.91,0.93)
1950 to 1959			0.94(0.94,0.95)
1955 to 1964			0.96(0.960.97)
1960 to 1969			0.98(0.97,0.98)
1965 to 1974			0.99(0.98,0.99)
1970 to 1979			1.00
1975 to 1984			1.00(1.00,1.01)
1980 to 1989			1.01(1.00,1.01)
1985 to 1994			1.01(1.01,1.02)
1990 to 1999			1.02(1.01,1.03)
1995 to 2004			1.04(1.03,1.05)

^a^Net drift: the overall annual percent change

^b^Local drift: Age group-specific annual percent change (%)

### The age, period and cohort effects on migraine incidence

**Fig. 1:**
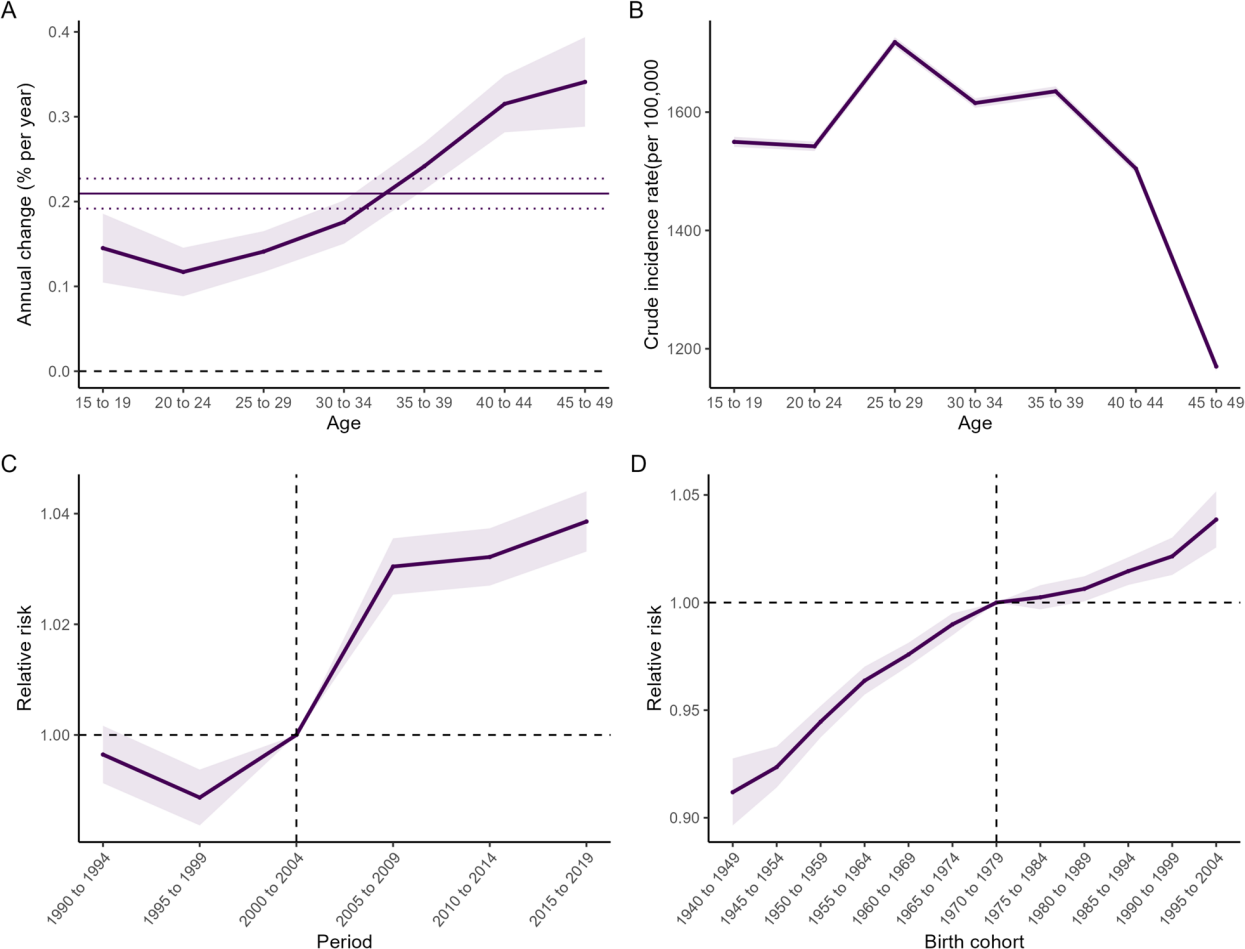
Relative risks of the incidence of Migraine in China from 1990 to 2019 due to effects of age, period, and cohort. **a** Net drifts (horizontal lines) and local drifts (curves). **b** Longitudinal age curves of migraine incidence in China. **c** Period effects on Migraine, with period 2000–2004 (median 2002) as reference period. **d** Cohort effects on Migraine, with cohort born in 1970–1979 (median 1975) as reference birth cohort

Figure 1 displayed the age, period and cohort effects on migraine incidence among women of childbearing age in China between 1990 and 2019. The net drift was estimated as 0.21% (95% CI: 0.19%, 0.23%) per year among overall subjects, while the lowest value for local drift (0.12%; 95% CI: 0.09%, 0.15%) was observed significant in the group aged 20–24 years (Fig. 1a). With period and cohort considered, the age-specific relative risks of migraine followed a bimodal pattern with peaks at the age-group of 25–29 years (CIR = 1718.27/100000; 95% CI: 1709.95/100000, 1726.63/100000) and 35–39 years (CIR = 1635.18/100000; 95% CI: 1626.83/100000, 1643.57/100000) (Fig. 1b). After control for age and cohort, the period-specific relative risks initially decreased from 1.00 (95% CI: 0.99, 1.00) in the 1990–1994 cohort to 0.99 (95% CI: 0.98, 0.99) in the 1995–1999 cohort, and then increased to 1.04 (95% CI: 1.03, 1.04) in the 2015–2019 cohort (Fig. 1c). With adjustment for age and period, the cohort-specific relative risks of migraine increased constantly from 0.91 (95% CI: 0.90, 0.93) in the 1940–1949 cohort to 1.04 (95% CI: 1.03, 1.05) in the 1995–2004 cohort (Fig. 1d).

### Migraine incidence projected for 2030

**Fig. 2:**
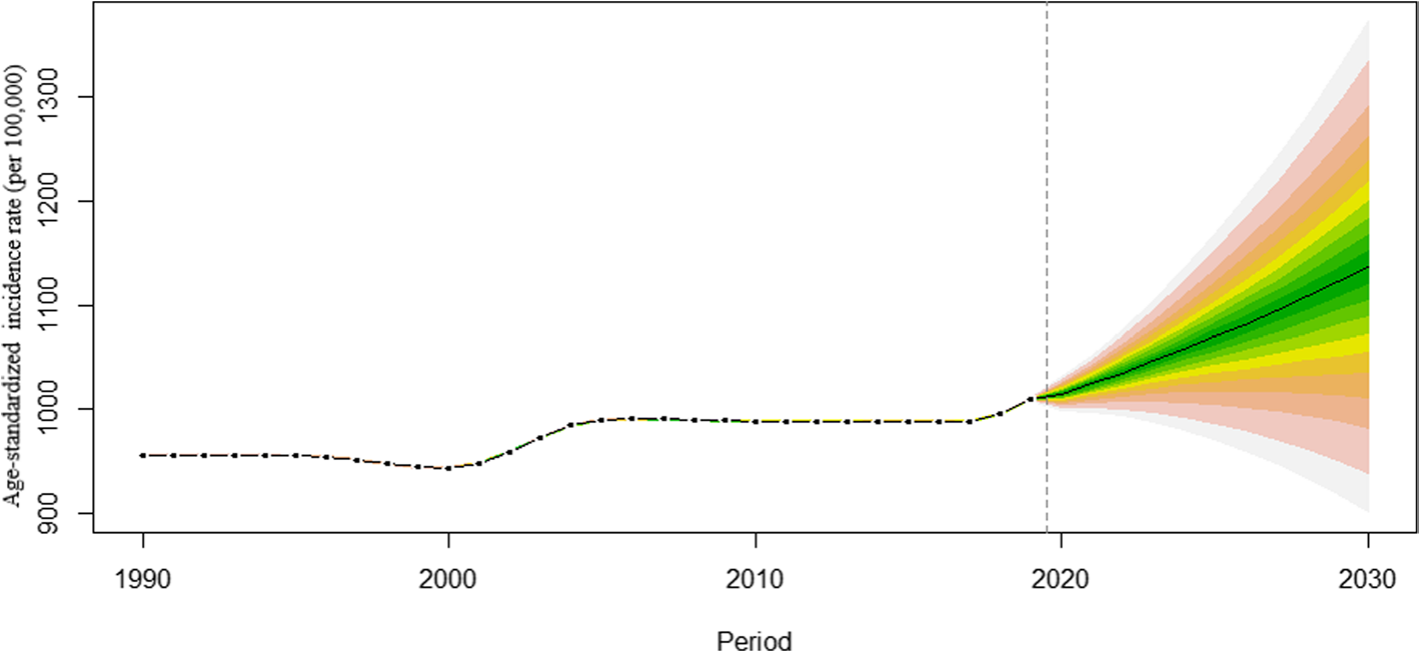
Forecast of Migraine ASIR (per 100,000) from 2020–2030 through Age-Period-Cohort Analysis

**Fig. 3:**
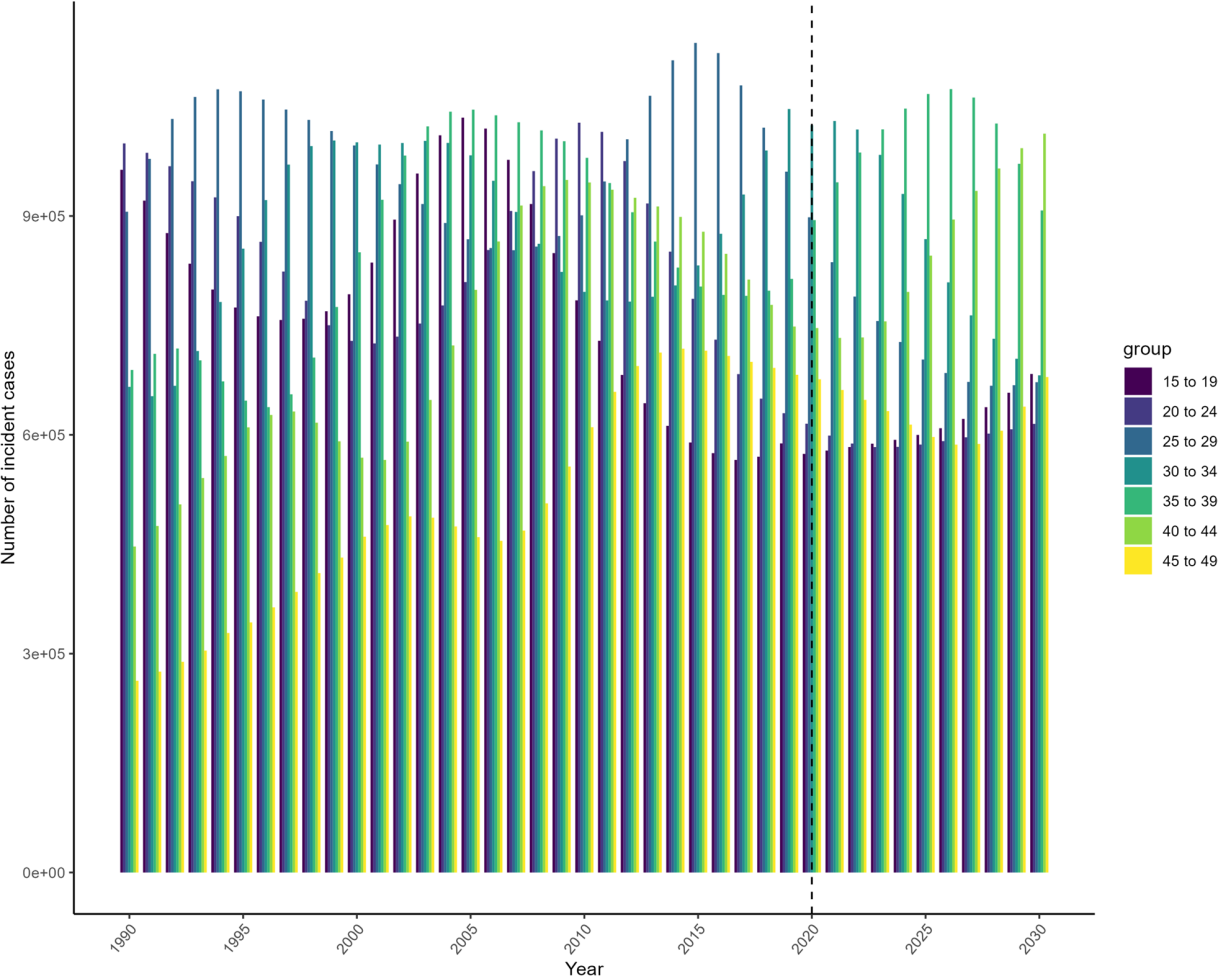
Trends in number of incidence migraine cases of migraine by age group from 1990 to 2030

**Fig. 4:**
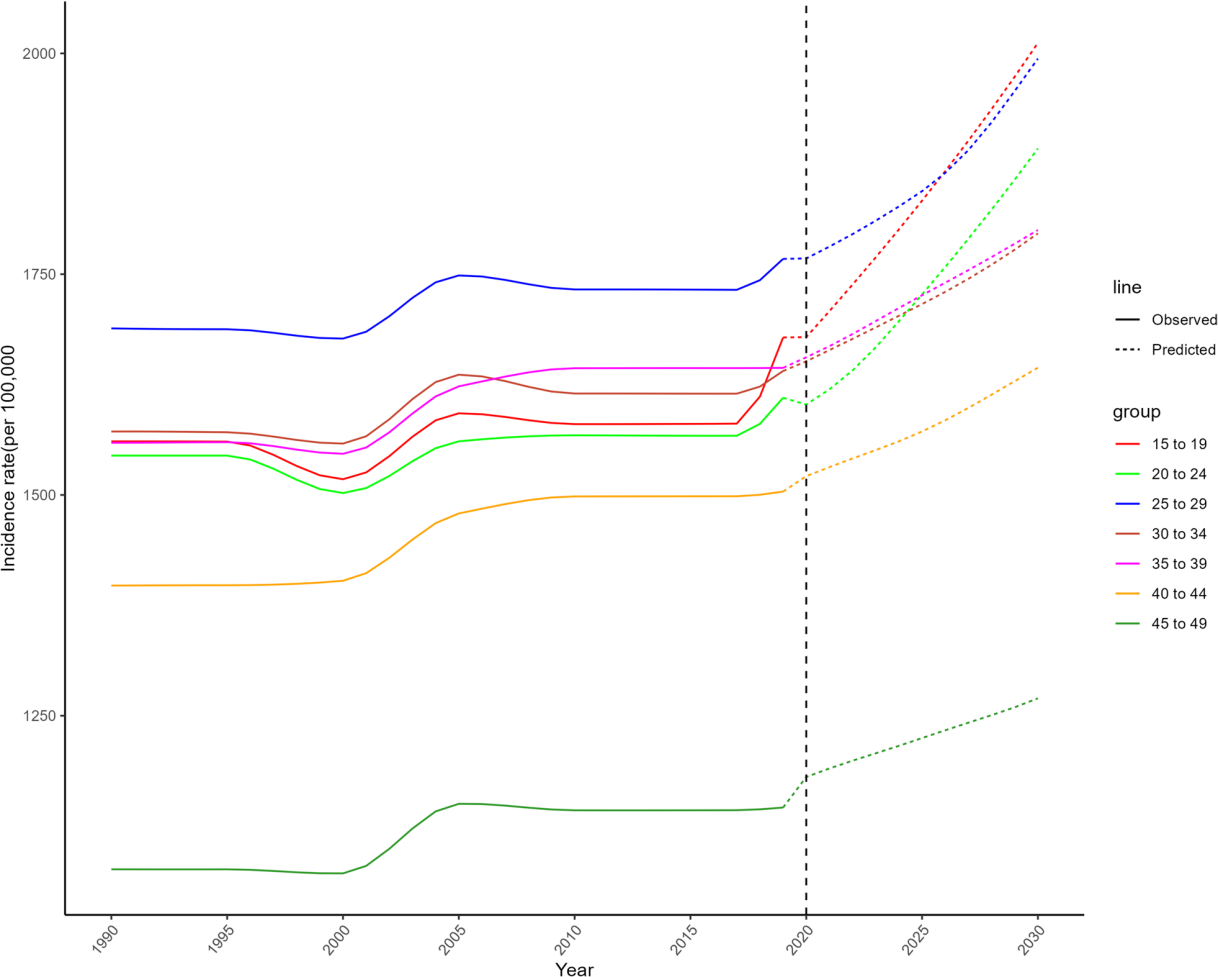
Trends in crude incidence rates of migraine by age group from 1990 to 2030

Table 4 demonstrated the cases, CIR and ASIR of migraine predicted for 2030 among women of childbearing age in China. It was estimated that CIR and ASIR of migraine would rise markedly from 1557.81/100,000 and 1590.29/100,000 in 2019 to 1719.27/100,000 and 1790.20/100,000 in 2030, respectively, among overall women of childbearing age in China, although the number of migraine cases would decline from 546.94 × 10^4^ in 2019 to 522.05 × 10^4^ in 2030 (Fig. 2). A rising trend is projected in participants aged 35–39 years and 40–44 years, but a declining trend in other age-groups (Fig. 3). Notably, by 2030, the greatest increase (35.36%) in the number of migraine cases would be observed in women aged 40–44 years. Similarly, the CIR of migraine would rapidly increase across all age-groups from 2019 to 2030. In 2030, the highest CIR of migraine would boost to 2011.84 per 100,000 persons for women aged 15 to 19 years, followed by those aged 25 to 29 years with 1994.11 per 100,000 persons (Fig. 4).

**Table 4:** Cases and incidence rate of migraine predicted for 2030 among women of childbearing age in China

Year	Measures	Women of childbearing age							
		Overall	15–19 years	20–24 years	25–29 years	30–34 years	35–39 years	40–44 years	45–49 years
	Cases^a^	522.05	68.34	61.5	67.21	68.15	90.76	101.29	67.92
2030	CIR^b^	1719.27	2011.84	1892.28	1994.11	1796.14	1799.97	1644.08	1269.69
	ASIR^c^	1790.2	N/A	N/A	N/A	N/A	N/A	N/A	N/A
	Change in number (%)	-4.55	16.17	-2.33	-30.05	-34.88	11.54	35.36	-0.41
2020–2030	Change in CIR (%)	10.36	19.83	17.5	12.82	9.49	9.5	9.33	10.83
	Change in ASIR (%)	12.57	N/A	N/A	N/A	N/A	N/A	N/A	N/A

^a^Cases: the number of migraine cases (× 10^4^)

^b^*CIR* Crude incidence rate (/100,000)

^c^*ASIR* Age-standardized incidence rate (/100,000)

## Discussion

This study using the entire national data aimed to investigate migraine incidence and its trend among women of childbearing age over three decades from 1990 to 2019, and to make a prediction of migraine incidence to the next decade in China. It was observed that, from 1990 to 2019, there was an increasing trend in all migraine cases, CIR and ASIR among overall study population. Moreover, although the number of migraine cases is predicted to decrease from 2020 to 2030, the CIR and ASIR of migraine will remain rapidly rising among women of childbearing age in China. Additionally, the predicted CIR of migraine will be higher among women aged 15–29 years than that for their counterparts of other ages, with the highest among women aged 15–19 years in the year of 2030.

The trend in migraine cases and ASIR examined in our study was in line with that observed among not only the general Chinese population [16] but also the global population [24]. Migraine is a physiological and psychological problem associated with some environmental, social, behavioral, physical and psychological factors, including air pollution [25], unexpected body weight status [26], frequent use of electronic products [27], physical inactivity [28], anxiety/stress[29] and sleep disorders [30]. These influencing factors of migraine were shared by people worldwide, which might, at least partially, explain the consistent findings regarding migraine cases and ASIR between our study and the previous investigations. However, the trend of migraine CIR in this study was inconsistent with that observed in the general population in China [16]. This may be due to that the proportion of women of childbearing age within the total population of China decreased from 26.79% in 1990 to 22.86% in 2020 [31].

The values of CIR and ASIR in women of childbearing age in our study were higher than those in either the general population or the overall women in China. Such an inconsistency might be attributed to biological, emotional state or/and lifestyle factors. From a biological point of view, it was assumed that sex hormones, especially fluctuations in estrogen and progesterone, might play a key role in the pathogenesis of migraine [12]. Since the pain threshold was lower for women than men, women might be more vulnerable to migraine [32]. Additionally, stress, lifestyle and behavioral patterns were also well-known triggers for migraine [33], and women tended to be physically inactive and to experience stress due to the complicated pressure from work and family life [34].

Another interesting finding in our study was that the risk of migraine varied for participants within different age-groups from 1990 to 2019. Based on the age-period-cohort analysis, the highest risk of experiencing migraine due to age effect was identified in the group aged 25–29 years, followed by those aged 35–39 years over the past three decades. For females aged 25–29 years, they are at the stage of transition from school to work and face complex work and social relationships, economic pressures, stress and anxiety [35]. Similarly, women aged 35–39 years may be experiencing the midlife crises, and have to play dual roles of working and caring for family and children [36]. Under such a situation, they need to maintain highly energetic and positive emotional states all the time, which may have them to feel tired and/or anxious [36].

Moreover, the highest migraine CIR for 2030 is predicted in young women aged 15–19 years. This may be due to the highly competitive pressures faced by Chinese children/adolescents and traditional parenting style in China [36]. Participants aged 15–19 years have to face pressures of curricula study, physical and mental development challenges, particularly, during the period of puberty and menarche. These individuals have to do their best to obtain excellent academic performance as possible under a highly competitive situation, and, meanwhile, to face hormonal fluctuations [37].

In this study, either the birth cohort or period was examined to be associated with incidence of migraine. The relative risk for cohort effect increased monotonically from the 1940–1949 birth cohorts to the 1995–2004 cohorts. There are several factors that may contribute to the cohort effect on migraine incidence. It has been examined that environmental pollution, stress, physical inactivity, overuse of medications and electronic devices are all in negative relation to migraine [29, 32]. In recent years, due to rapid economic and social development, these risk factors of migraine have been becoming more prevalent in China [38, 39]. Thus, compared to earlier-born participants, later-born subjects are more likely to be exposed to these risk factors. Consequently, coupled with the cumulative effect of time, people born later tend to suffer from migraine.

Moreover, the period-specific relative risk continued increasing over the 30 years except for the period of 1994–1999. This might be explained by the following two reasons. On the one hand, more risk factors associated with migraine emerged in the century in China, e.g., negative life events (SARS endemic, earthquakes, etc.), social competition, and unhealthy behaviors and/or lifestyles (staying up late, lack of exercise, addiction to electronic products, etc.) [16], which might increase the risk for residents to experience migraine. On the other hand, with the rapid development of medical technology and elevated health consciousness of participants, the identification rate of migraine might be consequently improved [40].

Additionally, a constantly increasing trend was predicted in CIR and ASIR of migraine in the next decade from 2020 to 2030, while the number of migraine cases was projected to decrease by 4.55% over the same period. This may be due to the changes in Chinese population structure in the next 10 years. China has been under a way to rapidly-aging society. It has been estimated that the proportion of residents aged 65 + years will increase from 12.06% in 2020 to 18.21% in 2030 [41]. Contrarily, the proportion of women of childbearing age will decrease in the next decade in China [42].

For women of childbearing age in China, the burden caused by migraine was very heavy during 1990 and 1999 and such a situation would remain for 2030. Meanwhile, women within this age-group tends to experience migraine due to complicated biological (e.g., female sex hormone fluctuation), emotional (e.g., anxiety, depression), lifestyle factors (e.g., sleep) or/and social factors (e.g., career competitiveness, family and child care) [12, 32–36]. Moreover, women of childbearing age also play critical and multiple roles not only in a society but also in each single family. The health state of each woman of childbearing age is really crucial for herself, her family and the society. Therefore, it is of particular importance and urgent necessity to initiate precision interventions on migraine among women of childbearing age in China.

### Strengths and limitations

This is the first study to comprehensively assess the incidence of and trend in migraine among women of childbearing age over a 30-year period using nationwide data in China. There were several strengths in this study. First, the definition and identification approach of migraine were adopted from those in GBD, which warranted the data regarding migraine were comparable across GBD-related studies [22]. Second, participants were the vulnerable sub-population for this disease, nationwide women of childbearing age. Third, the trend in migraine incidence over the past three decades was examined and a prediction for the next 10 years was projected. The last, the effects of age, period, and cohort on migraine incidence from a historical epidemiological perspective were estimated using the APC model among participants in the study.

However, limitations are also worthy of attention. Firstly, the definition and identification of migraine in our study were directly derived from GBD study, implying that the potential bias in GBD study also existed in our study [1, 19]. Secondly, very few influencing factors were included in GBD study, which did not allow us further identifying more factors in relation to the trend in migraine over the 30 years. Thirdly, data on migraine incidence were available only at national level, thus we had to just present the nationwide trend in migraine incidence. In future, well-designed population-level epidemiological surveys are encouraged to collect data on migraine, including its risk factors and participant’s personal characteristics, from a representative population.

## Conclusions

In conclusion, an increasing trend in migraine incidence has been observed from 1990 to 2019 and the migraine incidence will remain this elevating trend for 2030 among women of childbearing age in China. Participants’ age, period and birth cohort all contribute to the occurrence of migraine. This study has important public health implications for population-level migraine prevention in China. Precision intervention strategies and approaches shall be considered in campaigns initiated for migraine prevention among women of childbearing age.

## Data Availability

All the data involved in this work can be available from GBD 2019 study at http://ghdx.healthdata.org. Data Source: Institute for Health Metrics and Evaluation. Used with permission. All rights reserved.

## Abbreviations

GBD: The global burden of diseases, injuries, and risk factors study
ICHD: International classification of headache diseases
CIR: Crude incidence rate
ASIR: Age-standardized incidence rate
RR: Relative risk
APC: Annual percent change
AAPC: Average annual percent change

## References

[1] GBD 2019 Diseases and Injuries Collaborators. Global burden of 369 diseases and injuries in 204 countries and territories, 1990–2019: a systematic analysis for the Global Burden of Disease Study 2019. Lancet 2020; 396(10258): 1204–1222. 10.1016/S0140-6736(20)30925-9 10.1016/S0140-6736(20)30925-9 PMC7567026 33069326

[2] Leonardi M, Raggi A (2019). A narrative review on the burden of migraine: when the burden is the impact on people's life. J Headache Pain.

[3] Jeyagurunathan A, Abdin E, Vaingankar JA, Chua BY, Shafie S, Chang SHS (2020). Prevalence and comorbidity of migraine headache: results from the Singapore Mental Health Study 2016. Soc Psychiatry Psychiatr Epidemiol.

[4] Wang X, Xing Y, Sun J, Zhou H, Yu H, Zhao Y (2016). Prevalence, associated factors, and impact on quality of life of migraine in a community in northeast China. J Oral Facial Pain Headache.

[5] Foti M, Lo Buono V, Corallo F, Palmeri R, Bramanti P, Marino S (2017). Neuropsychological assessment in migraine patients: a descriptive review on cognitive implications. Neurol Sci.

[6] Gu X, Xie Y (2018). Migraine attacks among medical students in Soochow University, Southeast China: a cross-sectional study. J Pain Res.

[7] Yu S, Zhang Y, Yao Y, Cao H (2020). Migraine treatment and healthcare costs: retrospective analysis of the China health insurance research association (CHIRA) database. J Headache Pain.

[8] Steiner TJ, Stovner LJ, Jensen R, Uluduz D, Katsarava Z (2020). Lifting The Burden: the Global Campaign against Headache Migraine remains second among the world’s causes of disability, and first among young women: findings from GBD2019. J Headache Pain.

[9] Ashina M, Katsarava Z, Do TP, Buse DC, Pozo-Rosich P, Özge A (2021). Migraine: epidemiology and systems of care. Lancet.

[10] Lin QF, Xia QQ, Zeng YL, Wu XY, Ye LF, Yao LT (2018). Prevalence of migraine in Han Chinese of Fujian province: An epidemiological study. Medicine (Baltimore).

[11] Takeshima T, Wan Q, Zhang Y, Komori M, Stretton S, Rajan N (2019). Prevalence, burden, and clinical management of migraine in China, Japan, and South Korea: a comprehensive review of the literature. J Headache Pain.

[12] Vetvik KG, MacGregor EA (2017). Sex differences in the epidemiology, clinical features, and pathophysiology of migraine. Lancet Neurol.

[13] Ahmad SR, Rosendale N (2022). Sex and Gender Considerations in Episodic Migraine. Curr Pain Headache Rep.

[14] Buse DC, Fanning KM, Reed ML, Murray S, Dumas PK, Adams AM (2019). Life with migraine: effects on relationships, career, and finances from the chronic migraine epidemiology and outcomes (CaMEO) Study. Headache.

[15] Ishii R, Schwedt TJ, Kim SK, Dumkrieger G, Chong CD, Dodick DW (2020). Effect of migraine on pregnancy planning: insights from the American registry for migraine research. Mayo Clin Proc.

[16] Wang Y, Huang X, Yue S, Liu J, Li S, Ma H (2022). Secular Trends in the Incidence of Migraine in China from 1990 to 2019: A joinpoint and age-period-cohort analysis. J Pain Res.

[17] Yao C, Wang Y, Wang L, Liu Y, Liu J, Qi J (2019). Burden of headache disorders in China, 1990–2017: findings from the global burden of disease study 2017. J Headache Pain.

[18] Yang H, Pu S, Lu Y, Luo W, Zhao J, Liu E (2022). Migraine among students of a medical college in western China: a cross-sectional study. Eur J Med Res.

[19] GBD 2019 Risk Factors Collaborators. Global burden of 87 risk factors in 204 countries and territories, 1990–2019: a systematic analysis for the Global Burden of Disease Study 2019. Lancet 2020; 396(10258): 1223–1249. 10.1016/S0140-6736(20)30752-2 10.1016/S0140-6736(20)30752-2 PMC7566194 33069327

[20] GBD 2019 Demographics Collaborators. Global age-sex-specific fertility, mortality, healthy life expectancy (HALE), and population estimates in 204 countries and territories, 1950–2019: a comprehensive demographic analysis for the Global Burden of Disease Study 2019. Lancet 2020; 396(10258): 1160–1203. 10.1016/S0140-6736(20)30977-6 10.1016/S0140-6736(20)30977-6 PMC7566045 33069325

[21] GBD 2019 study. Available at: https://ghdx.healthdata.org/gbd-results-tool. Accessed on: April 20, 2023.

[22] Wei J, Chen L, Huang S, Li Y, Zheng J, Cheng Z (2022). Time trends in the incidence of spinal pain in China, 1990 to 2019 and Its prediction to 2030: the global burden of disease study 2019. Pain Ther.

[23] O'Brien, RM. Age-period-cohort models: Approaches and analyses with aggregate data. 2014. Available at: http://www.crcnetbase.com/isbn/9781466551541. Accessed on: April 20, 2023.

[24] Fan L, Wu Y, Wei J, Xia F, Cai Y, Zhang S (2023). Global, regional, and national time trends in incidence for migraine, from 1990 to 2019: an age-period-cohort analysis for the GBD 2019. J Headache Pain.

[25] Jiang X, Wang R, Chang T, Zhang Y, Zheng K, Wan R (2023). Effect of short-term air pollution exposure on migraine: A protocol for systematic review and meta-analysis on human observational studies. Environ Int.

[26] Huang Q, Liang X, Wang S, Mu X (2018). Association between body mass index and migraine: a survey of adult population in China. Behav Neurol.

[27] Migraine MH (2016). Long screen time exposure could increase the risk of migraine. Nat Rev Neurol.

[28] Lebedeva ER, Kobzeva NR, Gilev DV, Olesen J (2016). Factors associated with primary headache according to diagnosis, sex, and social group. Headache.

[29] Slatculescu AM, Chen Y (2018). Synergism between female gender and high levels of daily stress associated with migraine headaches in Ontario. Canada Neuroepidemiology.

[30] Kim J, Cho SJ, Kim WJ, Yang KI, Yun CH, Chu MK (2017). Insufficient sleep is prevalent among migraineurs: a population-based study. J Headache Pain.

[31] National Health Commission of The People’s Republic of China. China Health Statistic Yearbook 2022. Available at: http://www.nhc.gov.cn/mohwsbwstjxxzx/tjtjnj/202305/6ef68aac6bd14c1eb9375e01a0faa1fb.shtml. Accessed on: May 26, 2023

[32] Loewendorf AI, Matynia A, Saribekyan H, Gross N, Csete M, Harrington M (2016). Roads less traveled: sexual dimorphism and mast cell contributions to migraine pathology. Front Immunol.

[33] Marmura, Michael J. Triggers, Protectors, and Predictors in Episodic Migraine. Curr Pain Headache Rep. 2018; 22: 1–9. 10.1007/s11916-018-0734-0 10.1007/s11916-018-0734-0 30291562

[34] Griep RH, Toivanen S, Santos IS, Rotenberg L, Juvanhol LL, Goulart AC (2016). Work-family conflict, lack of time for personal care and leisure, and job strain in migraine: Results of the Brazilian Longitudinal Study of Adult Health (ELSA-Brasil). Am J Ind Med.

[35] Tan PL (2021). Stress, Fatigue, and Sexual Spontaneity Among Married Couples in a High-Stress Society: Evidence from Sex Diary Data from Singapore. Arch Sex Behav.

[36] He J, Ouyang F, Li L, Qiu D, Li Y, Xiao S (2021). Incidence trends of major depressive disorder in China: an age-period-cohort modeling study. J Affect Disord.

[37] Lin LY, Sidani JE, Shensa A, Radovic A, Miller E, Colditz JB (2016). Association between social media use and depression among US young adults. Depress Anxiety..

[38] Zhang M, Ma Y, Xie X, Sun M, Huang Z, Zhao Z (2023). Trends in insufficient physical activity among adults in China 2010–18: a population-based study. Int J Behav Nutr Phys Act..

[39] National Bureau of Statistics and Ministry of Environment Protection of China. China Environmental Statistical Yearbook, 2014, Table 8–19. http://www.stats.gov.cn/tjsj/ndsj/2014/indexeh.htm Available online accessed on: 13 September, 2015

[40] May A, Schulte LH (2016). Chronic migraine: risk factors, mechanisms and treatment. Nat Rev Neurol.

[41] Wei Y, Wang Z, Wang H, Li Y, Jiang Z (2019). Predicting population age structures of China, India, and Vietnam by 2030 based on compositional data. PLoS ONE.

[42] Chen W (2021). Declining number of births in China: a decomposition analysis. China popul dev stud.

